# What kinds of work do Japanese primary care physicians who derive greater positive meaning from work engage in? A cross‐sectional study

**DOI:** 10.1002/jgf2.595

**Published:** 2022-11-21

**Authors:** Yu Yamamoto, Junji Haruta, Ryohei Goto, Tetsuhiro Maeno

**Affiliations:** ^1^ General Medicine and Primary Care, Faculty of Medicine University of Tsukuba Ibaraki Japan; ^2^ Central General Clinic Ibaraki Japan; ^3^ Medical Education Center, School of Medicine Keio University Tokyo Japan; ^4^ Department of Primary Care and Medical Education, Faculty of Medicine University of Tsukuba Ibaraki Japan

**Keywords:** family medicine, research

## Abstract

**Background:**

Despite the increasing need for primary care physicians (PCPs) around the world, few physicians choose it as a career. PCPs who can find meaning and enjoyment in their work can be role models for medical students and professionals, which may encourage more physicians to specialize in primary care. We aimed to compare the kinds of work that Japanese PCPs who derive greater positive meaning from work engage in versus those who derive less positive meaning from work.

**Methods:**

This was a cross‐sectional study that used self‐administered questionnaires to ask Japanese PCPs about their basic characteristics and engagement in and enthusiasm for various types of work. The outcomes of the Japanese version of the work as meaning inventory (J‐WAMI) were also assessed. Participants were divided into high‐ and low‐scoring groups according to the median J‐WAMI score, and logistic regression analysis was performed to identify factors related to the high J‐WAMI‐scoring group.

**Results:**

A total of 268 out of 330 participants were included in the analysis. Males comprised 74.3%, and participants' average experience as a physician was 20.2 years. The median overall J‐WAMI score was 38. Factors associated with the high J‐WAMI scoring group were enthusiasm for outpatient care (OR: 1.04, 95% CI: 1.02–1.06) and engagement in research (OR: 2.74, 95% CI 1.33–5.66).

**Conclusions:**

Enthusiasm for outpatient care and engagement in research are associated with greater positive meaning of work among PCPs. Supporting these types of work may enhance PCPs' value of their work.

## Graphical abstract

Enthusiasm for outpatient care and engagement in research are associated with greater positive meaning of work among primary care physicians in Japan.

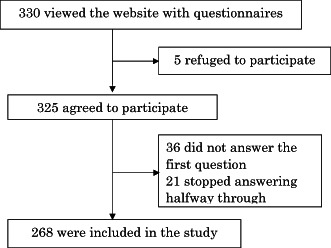

## 1 INTRODUCTION

Increasingly complex medical needs such as chronic diseases caused by aging, changing lifestyles, and diseases related to mental health highlight the importance of primary care around the world.^1^ In Japan, as in other countries, there is a rising need for primary care physicians (PCPs) who can provide more comprehensive and continuous care, as opposed to that offered through the limited lens of specific organs or diseases.^2^ PCPs are known by various names including general practitioners (GP) and family physicians (FP) in Japan. In this paper, we refer to them as PCPs.

To meet the increasing need for primary care, there has been a recent move towards training specialists as GP/FP. Unlike in Western countries, there was no system for certifying GP or FP in Japan for a long time; instead, organ‐specific specialists provided primary care.^3^ However, in 2010, the Japan Primary Care Association (JPCA) began certifying primary care specialists, and its training system obtained international certification from the World Organization of Family Doctors (WONCA) in 2020.^4^ In the intervening period, the Japanese Medical Specialty Board established a new specialist system in 2018.^3^ Today, it is difficult to determine the exact number of Japanese PCPs because of the characteristics of Japanese primary care, such as the fact that organ‐specific specialists provide primary care and that primary care is provided in both clinics and the outpatient departments of small‐ and medium‐sized hospitals. Available statistics indicate that there are 1067 JPCA‐certified family physicians, and 5349 with diplomas in primary care from JPCA (as of February 2022),^5^ and that 102,457 physicians work in clinics.^3^ As mentioned above, a training system for primary care is gradually being established,^3^ however, PCPs remain insufficiently recognized among medical professionals and medical students, and few physicians choose to specialize in primary care.^6^

There are several barriers and promoters for residents choosing primary care as a career. Barriers include a lack of local role models, anxiety related to dealing with diverse and broad areas, and uncertainty about the future.^7^ These barriers are thought to be linked to a lack of recognition of primary care by society and other departments, and few opportunities to meet PCP role models.^7^, ^8^ In particular, during clinical clerkships at university and in training before choosing a specialty, medical students rarely see PCPs active in the community, including in actual outpatient care or community activities, which may prevent them from realizing the attractiveness of primary care medicine.^7^ On the other hand, positive exposure to PCPs working in the field is an important promoter for the recruitment of students and residents.^9^ Additionally, when physicians enjoy and are satisfied with their work, this triggers the interests of medical students in the same field and has a significant impact on students' career choices.^10^ Medical students have been shown to be inspired by their role models to choose specialties they might not otherwise have considered.^10^ Thus, PCPs who enjoy their work may become role models for medical professionals and students, thereby increasing recognition of primary care and the number of physicians who specialize in primary care.^8^, ^9^, ^10^

One factor associated with enjoying and being satisfied in one's work is meaning of work (MW). The concept of MW has evolved in the career, management and psychological fields since the 1980 s.^11^ Although MW has a variety of definitions, we have adopted the original definition of “the central importance of work for people”.^12^ Several studies have found moderate or higher correlations between job satisfaction and MW, indicating that these concepts are closely related.^13^, ^14^ MW is also correlated with work engagement, commitment, life satisfaction, meaning of life, and general health measures.^13^ Thus, finding MW can lead to being satisfied with, enjoying and feeling enthusiastic about work, and greater general well‐being. Reports from outside Japan indicate that PCPs consider diagnosing and treating common diseases, following‐up on chronic diseases, and managing risk conditions to be meaning tasks.^15^, ^16^ However, there is little other research on MW among PCPs.

The purpose of this study was to compare the kinds of work that Japanese PCPs who derive greater positive meaning from work engage in versus those who derive less positive meaning from their work. This information may help PCPs find opportunities to develop meaning, enjoyment and satisfaction in their work.

## 2 METHODS

### 2.1 Design and participants

We conducted a cross sectional online study using self‐administered questionnaires. Based on the purpose of this study, participants were physicians who were members of JPCA, the largest academic organization related to primary care in Japan. Many physicians in the JPCA serve as role models for medical students and residents. Inclusion of JPCA members assumes that they agree with the principles of primary care regardless of the participants' original specialty. The exclusion criteria were junior residents defined as physicians, who graduated less than 2 years prior, and physicians who did not complete the questionnaire. We recruited participants through the JPCA mailing list from January 9 to February 12, 2021. The first page of the questionnaire provided a short description of the background and purpose of this study. Participants were informed that submission of the anonymous questionnaire implied that they were providing consent for participation. To calculate the required sample size, we considered that we would conduct a logistic regression analysis using around 10 independent variables, based on previous studies.^15^, ^16^, ^17^, ^18^ There would be dependent variables stratified by the median of the outcome values described below, and we assumed that the number of participants in each group would be similar. As at least ten participants per independent variable were required for each group,^19^ the sample size was calculated to be one hundred per each group, or two hundred overall.

### 2.2 Measures

The questionnaires inquired about the following participant characteristics: gender, number of years of experience as a physician, family structure (whether or not they live with a partner or children), whether or not they are JPCA‐certified family physicians, the size of the city where the physician works (large city with a population of 500,000 or more; medium city with a population of 100,000 to 300,000; small city with a population of around 50,000; village; and remote island and remote area), type of facility in which the physician works (clinic or hospital), and acceptance of students and residents for educational purposes at the facility where the physician works.

The outcome of this study, MW, was assessed using the Japanese version of The Work as Meaning Inventory (J‐WAMI). WAMI was developed by Steger et al. in 2012 to measure MW among individuals (Appendix S1).^20^ The scale theoretically explores the elements of MW, defining it through three subscales: positive meaning (PM); meaning‐making through work (MM); and greater good motivation (GG). Example items include “I have found a meaningful career” (PM), “I view my work as contributing to my personal growth” (MM), and “The work I do serves a greater purpose” (GG). The questionnaire contains 10 items. Each question is answered on a 5‐point Likert Scale, with 1 point indicating “absolutely untrue” and 5 points indicating “absolutely true”. Urata et al. developed J‐WAMI in 2019, and confirmed its reliability and validity.^21^ In this study, higher J‐WAMI scores were operationally defined as feeling more positive meaning of work.

The questionnaires also inquired about whether or not the participant had been engaged in clinical practice (outpatient care, home visit care, inpatient care, and emergency care), education, research, community activities, and meetings and management in the past year. We used engagement in each type of work as an objective indicator in the analysis. The participants were also asked to rate their enthusiasm for clinical practice (outpatient care, home visit care, inpatient care, emergency care), education, research, community activities, and meetings and management from 0 to 100 on a visual analog scale. For jobs in which they were not currently engaged, the participants were asked how enthusiastic they would be about becoming engaged in each job if they had the opportunity to do so in the future. Physicians' enthusiasm for each type of work was used as a subjective index in the analysis.

### 2.3 Data analysis

J‐WAMI scores are presented as median and interquartile range (IQR), while other continuous variables are presented as mean and standard deviation (SD). To identify the characteristics of those with high J‐WAMI scores, participants were divided into high and low J‐WAMI scoring groups based on a cutoff. Given the paucity of related studies, we used the median, or mean, as the cutoff. In univariate analysis, differences between the two groups were analyzed using a *t*‐test for continuous variables and chi‐squared test or Fisher's exact test for categorical variables. Variables found to be moderately associated with the high‐scoring group (*p* < 0.1) were further analyzed using logistic regression analysis. Gender and years of experience as a physician were added as independent variables to those found to be significantly correlated with the high‐scoring group in the univariate analysis. *p* < 0.05 was used for tests of statistical significance. In this study, we performed a complete case analysis. All analyses were performed using SPSS version 27 (IBM). Our study matched the STROBE criteria (Appendix S2).

### 2.4 Ethical considerations

This study was conducted with the approval of the Medical Ethics Committee of the University of Tsukuba (approval number. 1601).

## 3 RESULTS

### 3.1 Descriptive statistics

**FIGURE 1:**
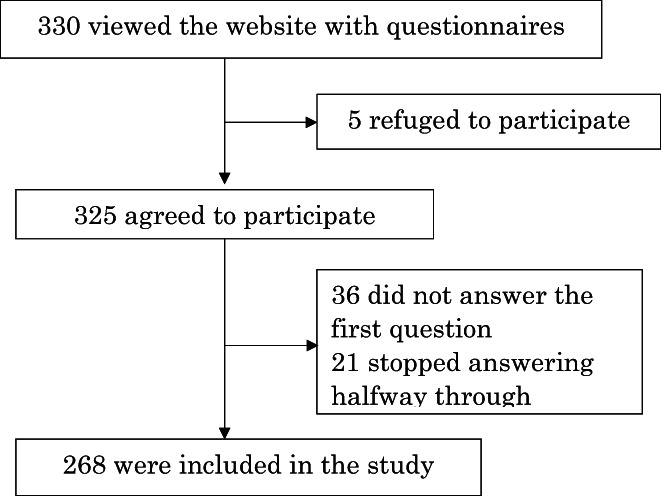
Flow chart of primary care physician participants in the study. Of the 330 participants who viewed the questionnaires, 268 physicians who agreed to participate and answered all questions were included in the analysis.

After two additional reminders and confirmation that the number of responses had reached the expected sample size, recruitment was closed. At the time the study was conducted, there were approximately 5500 physician members on the JPCA mailing list. Of the 330 participants who viewed the questionnaires, 268 physicians who agreed to participate and answered all questions were included in the analysis (Figure 1). Of the 268 physicians, 199 indicated they were male (74.3%), one indicated “other” and one did not provide a response. Participants' average experience as a physician was 20.2 years (SD 10.7), and 81 (30.2%) indicated they were JPCA‐certified family physicians. A total of 135 physicians (50.4%) reported that they were working in clinics, while one answered “other” (see the “overall” column in Table 1).

**TABLE 1:** Basic characteristics of participants and comparison between high and low J‐WAMI‐scoring groups

	Overall, *n* = 268	Low‐scoring group <38 points, *n* = 121	High‐scoring group ≥38 points, *n* = 147	*p*‐Value
Male, *n* (%)^b^	199 (74.3)	88 (72.7)	111 (75.6)	0.538
Years of experience as a physician, years, mean (SD)^a^	20.2 (10.7)	21.17 (10.0)	19.4 (11.0)	0.167
Family structure (including duplicates)				
Living with a partner, *n* (%)^b^	217 (81.0)	96 (79.3)	121 (82.3)	0.537
Living with children, *n* (%)^b^	157 (58.6)	63 (52.1)	94 (63.9)	0.049
JPCA‐certified family physician, *n* (%)^b^	81 (30.2)	36 (29.8)	45 (30.6)	0.879
Size of the city where the physician works				
Small city or smaller, *n* (%)^b^	96 (35.8)	44 (36.3)	52 (35.6)	0.866
Medium‐sized city or larger, *n* (%)^b^	172 (64.2)	77 (63.6)	95 (64.6)	
Type of facility in which the physician works				
Clinic, *n* (%)^b^	135 (50.4)	60 (49.6)	75 (51.0)	0.772
Hospital, *n* (%)^b^	132 (49.3)	61 (50.4)	71 (48.3)	
Acceptance of students, residents for educational purposes at the facility where the physician works, *n* (%)^b^	199 (74.3)	90 (74.3)	109 (74.1)	0.966

Abbreviations: JPCA, Japan Primary Care Association; J‐WAMI, Japanese version of the work as meaning inventory; SD, standard deviation.

^a^ *t*‐Test.

^b^ Chi‐squared test.

Because the total J‐WAMI score was not normally distributed in this study, participants were divided into two groups based on the median J‐WAMI score (38 points [IQR 34–41]): those with ≥38 points formed the high‐scoring group, and those with <38 points formed the low‐scoring group. The median J‐WAMI score was 41 [IQR 39–44] for the high‐scoring group (*n* = 147), and 34 [IQR 30–36] for the low‐scoring group (*n* = 121) (Table 2). Among the scale items, only item 2, “I view my work as contributing to my personal growth,” showed a ceiling effect.

**TABLE 2:** Total J‐WAMI score, engagement in and enthusiasm for different types of work and comparison between high‐ and low‐scoring groups

	Overall, *n* = 268	Low‐scoring group <38 points, *n* = 121	High‐scoring group ≥38 points, *n* = 147	*p*‐Value
Total J‐WAMI score^a^, median (IQR)^b^	38 (34–41)	34 (30–36)	41 (39–44)	<0.001
Subscale score^c^				
Positive meaning, median (IQR)^b^	16 (14–17)	14 (12–15)	17 (16–19)	<0.001
Meaning‐Making Through Work, median (IQR)^b^	12 (11–13)	10 (8–11)	13 (12–14)	<0.001
Greater Good Motivations, median (IQR)^b^	10 (9–12)	9 (7–10)	12 (11–12)	0.003
Engagement in each type of work (reference: no)				
Outpatient care, *n* (%)^d^	265 (98.9)	118 (97.5)	147 (100.0)	0.091
Home visit care, *n* (%)^e^	190 (70.9)	83 (68.6)	107 (72.8)	0.452
Inpatient care, *n* (%)^e^	124 (46.3)	57 (47.1)	67 (45.7)	0.803
Emergency care, *n* (%)^e^	130 (48.5)	61 (50.4)	69 (47.0)	0.571
Education, *n* (%)^e^	204 (76.1)	82 (67.8)	122 (83.0)	0.004
Research, *n* (%)^e^	136 (50.8)	48 (39.7)	88 (59.9)	<0.001
Community activities, *n* (%)^e^	178 (66.4)	74 (61.2)	104 (70.8)	0.098
Meetings and management, *n* (%)^e^	210 (78.4)	88 (72.7)	122 (83.0)	0.042
Enthusiasm for each type of work^f^				
Outpatient care, mean (SD)^b^	77.6 (19.8)	70.6 (22.4)	83.31 (15.1)	<0.001
Home visit care, mean (SD)^b^	67.0 (31.5)	60.3 (31.3)	72.6 (30.6)	0.001
Inpatient care, mean (SD)^b^	46.8 (35.2)	43.0 (35.9)	49.8 (34.5)	0.119
Emergency care, mean (SD)^b^	44.8 (32.0)	40.7 (31.5)	48.1 (32.2)	0.058
Education, mean (SD)^b^	59.5 (30.7)	51.2 (31.1)	66.4 (28.6)	<0.001
Research, mean (SD)^b^	42.2 (33.1)	34.4 (33.1)	48.7 (31.8)	<0.001
Community activities, mean (SD)^b^	59.7 (28.1)	52.5 (28.2)	65.6 (26.7)	<0.001
Meetings and management, mean (SD)^b^	47.3 (29.4)	41.7 (28.9)	51.8 (29.1)	0.005

Abbreviations: IQR, interquartile range; SD, standard deviation.

^a^ The maximum total J‐WAMI score is 50.

^b^ *t*‐Test.

^c^ The maximum subscale score in the J‐WAMI is 20 points for Positive Meaning, 15 points for Meaning‐Making Through Work, and 15 points for Greater Good Motivations.

^d^ Fisher's exact test.

^e^ Chi‐squared test.

^f^ Enthusiasm for each type of work was measured from 0 to 100 on a visual analog scale.

Regarding the physician's engagement in various types of clinical and nonclinical work in the past year, in terms of clinical work, 265 (98.9%) physicians indicated they were engaged in outpatient care, while 190 (70.9%) physicians conducted home visit care. Among nonclinical work, 136 (50.8%) physicians were engaged in research. Physicians' enthusiasm for clinical work was highest at 77.6 (SD 19.8) for outpatient care, and 67.0 (SD 31.5) for home visit care. In contrast, they indicated lower enthusiasm for emergency care and research at 44.8 (SD 32.0) and 42.2 (SD 33.1), respectively (see the “overall” column in Table 2).

### 3.2 Comparison of basic characteristics and each measure between high‐ and low‐scoring groups

We compared participants' basic characteristics, total J‐WAMI score, engagement in each type of work, and enthusiasm for each type work between the high‐ and low‐scoring groups. Because only a small number of respondents answered “other” for gender and type of work facility, they were excluded from the analysis. We confirmed that the high‐scoring group had significantly higher total J‐WAMI scores than the low‐scoring group (*p* < 0.01). In terms of basic characteristics, the high‐scoring group was significantly more likely to live with children (*p* = 0.049). In terms of engagement in each type of work, the high‐scoring group was more likely to be engaged in education, research, community activity and meetings and management (*p* = 0.091, *p* = 0.004, *p* < 0.001, *p* = 0.098, *p* = 0.042, respectively). In terms of enthusiasm for each type of work, the high‐scoring group was significantly more likely to be enthusiastic about outpatient care, home visit care, emergency care, education, research, community activities, and meetings and management (*p* < 0.001, *p* = 0.001, *p* = 0.058, *p* < 0.001, *p* < 0.001, *p* < 0.001, and *p* = 0.005, respectively) (see columns for the high‐scoring group and low‐scoring group in Tables 1 and 2).

### 3.3 Factors associated with the high‐scoring group

Based on the logistic regression analysis, the high‐scoring group was associated with enthusiasm for outpatient care (odds ratio [OR]: 1.04, 95% confidence interval [CI] 1.02 to 1.06) and engagement in research (OR: 2.85, 95% CI 1.39–5.84) (Table 3). All variance inflation factor values were <2.2, and no obvious multicollinearity was identified.^22^

**TABLE 3:** Results of logistic regression analysis of the factors associated with the high‐scoring group

	OR of high scoring group	95% CI	*p*‐Value
Years of experience as a physician	0.99	0.96–1.02	0.376
Male (reference: female)	1.59	0.89–2.85	0.115
Living with children (reference: no)	1.43	0.81–2.52	0.219
Engagement in education (reference: no)	1.53	0.73–3.21	0.25
Engagement in research (reference: no)	2.85	1.39–5	0.006
Engagement in community activity (reference: no)	1.31	0.69–2.46	0.409
Engagement in meetings and management (reference: no)	1.29	0.63–2.65	0.625
Enthusiasm for outpatient care^a^	1.04	1.02–1.06	<0.001
Enthusiasm for home visit care^a^	1.01	1.00–1.02	0.254
Enthusiasm for emergency care^a^	0.99	0.99–1.00	0.559
Enthusiasm for education^a^	1.00	0.99–1.02	0.366
Enthusiasm for research^a^	1.00	0.99–1.01	0.638
Enthusiasm for community activities^a^	1.01	0.99–1.02	0.375
Enthusiasm for meetings and management^a^	1.00	0.99–1.01	0.988

Abbreviations: CI, confidence interval; OR, odds ratio.

^a^ Enthusiasm was measured from 0 to 100 on a visual analog scale.

## 4 DISCUSSION

We found that Japanese PCPs who derive greater positive meaning from their work were more enthusiastic about outpatient care and engaged in research.

### 4.1 Relationship between enthusiasm for outpatient care and meaning of work

While few studies have examined MW among PCPs, there are some reports on job satisfaction, rewards, and identity among this profession. Studies on job satisfaction among PCPs report a relationship with variety in clinical practice, successful medical management of patients and the subsequent feeling of being competent, bonds with patients and family members, and passion for the work.^18^, ^23^ Another study reported that a PCP's identity is linked to a balance in the breadth and depth of clinical practice, patient/family relationships, and the comprehensive nature of patient care.^24^

In primary care outpatient settings in Japan, about 90% of PCPs routinely address various health problems, from internal medicine to musculoskeletal and dermatological fields.^17^ They also have to cope with multimorbidity, an increasing issue in the aging population,^25^ and with complex problems that require a psychosocial approach.^26^ Thus, for PCPs, outpatient care may be an area that requires improvisation, which involves creativity, to manage a wide range of ages, problems and disease stages; provide long‐term and relational continuity; deal with the complexity and comprehensiveness of the problem.^27^ Creativity is one factor that has been found to differ between PCPs and internists in previous studies on factors related to job satisfaction.^28^ In other words, primary outpatient care is one area where PCPs can exercise their role by performing the creative work required to address undifferentiated and varied problems in a comprehensive approach. Physicians who are able to enthusiastically engage in outpatient care are likely to have a higher sense of their role as a PCP, and will consequently consider their work more meaningful. In addition, it is easier to observe positive changes in patients after physician interventions in outpatient care, and repeated exposure to such positive changes may enhance physicians' MW. However, there are reports that PCPs who care for patients with multimorbidity are likely to experience burnout.^29^ Younger physicians and others with inadequate skills in outpatient care should be mindful of burnout, as it may reduce their enthusiasm for providing outpatient care resulting from the burden of treating highly complex patients.

### 4.2 Relationship between research engagement and MW

Few studies have examined the relationship between research engagement and job satisfaction or other aspects of PCPs' work. Previous studies have noted the low level of research in primary care in Japan.^30^ In this study, about half of the physicians responded that they were engaged in research. It is possible that there was selection bias in our study, in that the physician participants may have already had an interest and engagement in research. Our use of a broader definition of the term “research” in this study, which included writing case reports and providing support for conference presentations, may also have led many respondents to indicate they were engaged in research.

In this study, almost all of the participants who indicated they routinely conducted research were engaged in outpatient care and were clinician researchers^31^ who were engaged in both clinical practice and research. In Japan, physicians are often assigned clinical duties even in research and educational institutions such as universities. Clinician researchers can address emerging and relevant real‐world issues that arise in daily practice while accounting for the actual situation in the field, and promote evidence‐based practice, thus acting as a bridge between the research and practice worlds.^31^ Thus, the participants' research achievements and ability to translate their findings to patients and the community as clinicians may underlie their sense of accomplishment and enhanced MW. Furthermore, research in the primary care field covers a wide range of topics^32^, ^33^ and aims to solve problems at the individual level as well as the family, social and community levels.^31^, ^34^, ^35^ Thus, PCPs may feel that they are contributing to the community and society more broadly through their research, which may make their work feel more meaningful.

### 4.3 Impact on future practice and research

As this was a cross‐sectional study, we could not determine a strict causal relationship between MW and having enthusiasm for outpatient care or being engaged in research. Past studies have reported that work becomes meaningful when people are able to demonstrate competence in an area that they feel is their identity, when they are able to positively impact someone or something other than themselves, when they are challenged and accomplish a task, and when they are doing work that is important to society.^11^ Therefore, it is possible that having enthusiasm for outpatient care and being engaged in research can lead PCPs to derive positive meaning from their work through the experiences described above. The finding that enthusiasm for outpatient care is associated with positive MW among PCPs could prompt more active engagement in outpatient care, which tends to be a routine task. Furthermore, the result suggests that developing support strategies to encourage engagement in outpatient care may also be important. PCPs who are enthusiastic about outpatient care can also be role models to medical students and residents, and improving education for residents who find outpatient care burdensome may help enhance the careers of PCPs. In addition, for those interested in research, an environment that allows them to focus on research or a structure that facilitates participation in research from their residency may extend their engagement in this type of work.^36^

While most previous studies have been concerned with investigating factors that have a positive impact on MW, recent work has increasingly focused on the negative aspects of MW.^14^ Future research that also examines factors that reduce MW, such as burnout, which is a common occurrence in PCPs,^37^ may offer important insights.

### 4.4 Limitations

There were several limitations in this study. First, while WAMI has been used in some studies on PCPs, it has not been validated in healthcare settings or among PCPs. Therefore, the concept assessed by WAMI may not be fully compatible with Japanese PCPs. However, this questionnaire incorporates the abstract concept of MW into 10 concrete questions and could be useful in future research once validated. Further research is also needed to determine the optimal way to set cutoff values for the J‐WAMI score using a larger sample size and range of participants. Second, because we conducted a web‐based study, there may have been selection bias in that respondents may have already had a strong interest in the topic of this survey and high baseline MW. Additional research using tools other than web‐based surveys is needed to confirm our findings. Third, not all Japanese PCPs are members of the JPCA. However, as we conducted this study with the intention of applying the findings to future specialty training programs, we chose to focus on JPCA members to include physicians who have received specialty training and are currently supervising physicians. Fourth, past studies have shown that MW is negatively associated with depression and anxiety. Because we did not assess these symptoms, the impact of depression and anxiety on MW should be more comprehensively assessed in the future. Fifth, caution is needed when generalizing the results because of the unique context of primary care in Japan. However, the results may be adaptable to Asian countries where primary care is still a developing field.^38^ Furthermore, given the scarcity of research on primary care worldwide, we believe our findings may provide useful background or a means for comparison in research in other countries.

## 5 CONCLUSION

Enthusiasm for outpatient care and engagement in research are associated with positive MW among PCPs. Supporting outpatient care, where PCPs can better exercise their identity, and research in the primary care field, which can serve as a bridge to the clinical world, may contribute to positive values like satisfaction and enjoyment among physicians through enhancing MW.

## FUNDING INFORMATION

This work was supported by JSPS KAKENHI Grant Number 21K10297.

## CONFLICT OF INTEREST

The authors have stated explicitly that there are no conflicts of interest in connection with this article.

## ETHICS APPROVAL

This research has been approved by the Ethics Committee of the University of Tsukuba (approval number: 1601).

## PATIENT CONSENT STATEMENT

All study participants agreed to participate in this study.

## CLINICAL TRIAL REGISTRATION

None.

## Supporting information


Appendix S1

Appendix S2

## Data Availability

The data underlying this article will be shared on reasonable request to the corresponding author.
