# Supplementary material for: What kinds of work do Japanese primary care physicians who derive greater positive meaning from work engage in? A cross‐sectional study
Source: J Gen Fam Med. 2022 Nov 21;24(2):94–101. doi: 10.1002/jgf2.595 (PMC10000278; doi:10.1002/jgf2.595)
Supplement: Supplementary file 1 — Appendix S1 [file JGF2-24-94-s002.pdf]

The Work and Meaning Inventory. Work can mean a lot of different things to different people. The following items ask about how you see the role of work in your own life. Please honestly indicate how true each statement is for you and your work.

	Absolutely Untrue	Mostly Untrue	Neither True nor Untrue	Mostly True	Absolutely True
1. I have found a meaningful career	1	2	3	4	5
2. I view my work as contributing to my personal growth.	1	2	3	4	5
3. My work really makes no difference to the world.	1	2	3	4	5
4. I understand how my work contributes to my life's meaning.	1	2	3	4	5
5. I have a good sense of what makes my job meaningful.	1	2	3	4	5
6. I know my work makes a positive difference in the world.	1	2	3	4	5
7. My work helps me better understand myself.	1	2	3	4	5
8. I have discovered work that has a satisfying purpose.	1	2	3	4	5
9. My work helps me make sense of the world around me.	1	2	3	4	5
10. The work I do serves a greater purpose.	1	2	3	4	5

© 2011 Michael F. Steger. The Work and Meaning Inventory (WAMI) can be used in research and educational capacities without restriction. Permission for commercial or revenue-generating applications of the WAMI must be obtained from Michael F. Steger prior to use.

Scoring instructions.

- Add the ratings for items 1, 4, 5, and 8 to get the "*Positive Meaning*" score. The *Positive Meaning* scale reflects the degree to which people find their work to hold personal meaning, significance, or purpose.

- Add the ratings for items 2, 7, and 9 to get the "*Meaning-Making through Work*" score. The *Meaning-Making through Work* score reflects the fact that work is often a source of broader meaning in life for people, helping them to make sense of their live experience.

- Subtract the rating for item 3 from 6 (e.g., if a client gave item 3 a rating of 2, then their converted rating would be 4 [6-2=4]); add this number to the ratings for items 6 and 10 to get the "*Greater Good Motivations*" score. The *Greater Good Motivations* score reflects the degree to which people see that their effort at work makes a positive contribution and benefits others or society.

- The *Positive Meaning, Meaning-Making through Work*, and *Greater Good Motivations* scores can all be added together to get the test-taker's overall **Meaningful Work** score. The **Meaningful Work** score reflects the depth to which people experience their work as meaningful, as something they are personally invested in, and which is a source of flourishing in their lives.

Low scores on any of these scales reflect an absence of work meaning, and may be predictive of poor work engagement, low commitment to one's organization and intentions to leave, low motivation, a perceived lack of support and adequate guidance from leadership or management. People who score low on these scales are also more likely to be absent from work and experience both low levels of well-being and higher levels of psychological distress.

For more information on the development of the WAMI, please consult:
Steger, M. F., Dik, B. J., Duffy, R. D. (in press). Measuring Meaningful Work: The Work and Meaning Inventory (WAMI). Journal of Career Assessment.

For a case vignette of how to use the WAMI with individual clients, please consult:
Steger, M. F., Dik, B. J., & Shim, Y. (in press). Assessing meaning and satisfaction at work. In S. J. Lopez (Ed.), The Oxford handbook of positive psychology assessment (2nd Ed.). Oxford, UK. Oxford University Press.

To contact the test developer, please contact *michael_f_steger@yahoo.com*.
